# Insulin autoimmune syndrome: case report

**DOI:** 10.1590/S1516-31802004000400010

**Published:** 2004-07-01

**Authors:** Rodrigo Oliveira Moreira, Giovanna Aparecida Balarini Lima, Patrícia Carla Batista Peixoto, Maria Lucia Fleiuss Farias, Mario Vaisman

**Keywords:** Hypoglycemia, Hyperinsulinism, Insulin, Pancreatectomy, Insulin antibodies, Hipoglicemia, Hiperinsulinismo, Insulina, Pancreatectomia, Anticorpos antiinsulina

## Abstract

**CONTEXT:**

Insulin autoimmune syndrome (IAS, Hirata disease) is a rare cause of hypoglycemia in Western countries. It is characterized by hypoglycemic episodes, elevated insulin levels, and positive insulin antibodies. Our objective is to report a case of IAS identified in South America.

**CASE REPORT:**

A 56-year-old Caucasian male patient started presenting neuroglycopenic symptoms during hospitalization due to severe trauma. Biochemical evaluation confirmed hypoglycemia and abnormally high levels of insulin. Conventional imaging examinations were negative for pancreatic tumor. Insulin antibodies were above the normal range. Clinical remission of the episodes was not achieved with verapamil and steroids. Thus, a subtotal pancreatectomy was performed due to the lack of response to conservative treatment and because immunosuppressants were contraindicated due to bacteremia. Histopathological examination revealed diffuse hypertrophy of beta cells. The patient continues to have high insulin levels but is almost free of hypoglycemic episodes.

## RESUMO

**CONTEXTO:**

A síndrome da hiperinsulinemia autoimune (SHA, doença de Hirata) é uma causa rara de hipoglicemia nos países ocidentais. Ela é caracterizada por episódios de hipoglicemia, níveis elevados de insulina e presença de anticorpos anti-insulina. Nosso objetivo é relatar um caso da SIA identificado na América do Sul.

**RELATO DO CASO:**

Um homem caucasiano de 56 anos de idade começou a apresentar sintomas neuroglicopênicos durante hospitalização devida a trauma grave. A avaliação laboratorial confirmou hipoglicemia e níveis extremamente elevados de insulina. Os exames radiológicos convencionais realizados foram negativos para tumor pancreático. A remissão clínica da doença não ocorreu durante o uso de verapamil e corticóides. Desta forma, pancreatectomia subtotal foi realizada devido à ausência de resposta ao tratamento conservador e à impossibilidade de serem utilizados imunosupressores em razão da bacteremia persistente. O exame histopatológico revelou hiperplasia difusa de células beta. O paciente persiste apresentando níveis elevados de insulina porém praticamente não apresenta mais episódios de hipoglicemia.

## INTRODUCTION

Insulin autoimmune syndrome (IAS, Hirata disease) is an unusual cause of hypoglycemia. It is characterized by spontaneous hypoglycemia, extremely high insulin levels, and the presence of circulating insulin antibodies in patients who have never been exposed to exogenous insulin. Although IAS is considered to be the third greatest cause of hypoglycemia in Japan, only a handful of cases have been reported in Western countries.^1^ Our objective is to report a case of IAS identified in South America.

## CASE REPORT

Mr. A, a 56-year-old retired Caucasian man, presented episodes of symptomatic hypoglycemia during hospitalization due to severe trauma. He was submitted to amputation of the right leg, developed severe sepsis, and had to be kept on wide-spectrum antibiotics. His treatment included dopamine, because of the septic shock, and continuous intravenous glucose for the prevention of hypoglycemia. Endogenous hyperinsulinemia was suspected, and this was confirmed by the patient's low glycemic level, elevated serum insulin (23710 μIU/ml, after 1/50 dilution of the serum), and high serum levels of C peptide (4.2 ng/ml). He said he had not been using exogenous insulin and was unaware of cases of hypoglycemia in his family.

At that moment, conventional imaging examinations were requested in an attempt to investigate the possibility of insulinoma. Abdominal ultrasound, computed tomography and pancreatic arteriography were negative. However, selective arterial calcium stimulation and hepatic venous sampling (ASVS) demonstrated high levels of insulin and C peptide in the whole pancreas and no response to calcium stimulation (Table 1).

**Table 1:** Insulin and C peptide levels during arterial stimulation and venous sampling (ASVS) in an adult patient with insulin autoimmune syndrome (insulin results were only available after 1/50 dilution of the serum)

	Basal		30 seconds		60 seconds		120 seconds	
Arteries	Insulin	C peptide	Insulin	C peptide	Insulin	C peptide	Insulin	C peptide
Hepatic	5795	4.3	7605	4.3	6395	4.5	7185	4.0
Distal splenic	3955	3.2	4005	3.6	4590	3.9	4895	3.7
Proximal splenic	5330	3.8	5170	3.6	5520	3.5	5050	3.9
Gastroduodenal	7035	4.3	5785	4.2	7865	4.2	6365	4.2
Superior mesenteric	7740	4.4	5555	4.3	6245	4.5	6090	4.4

*Reference values: insulin: 5 – 25 μIU/ml; C peptide: 0.8 – 4.0 ng/ml.*

During the preoperative period, verapamil and prednisone were prescribed without success. After five months in hospital, no signs of remission of hypoglycemia were detected. Surgery was indicated due to the severity of the episodes and because immunosuppressants were contraindicated due to bacteremia. During the operation, no tumor was found by palpation, thus prompting the decision to perform subtotal pancreatectomy. Histological examination demonstrated diffuse hyperplasia of beta cells.

Data on antibody levels were available only after the surgical procedure. The levels of islet cell antibody (ICA) and glutamic acid decarboxylase antibody (anti-GAD) were within normal range. Insulin auto-antibodies (IAA) were elevated (107.30 IU/ml; the reference range is from 0.00 to 1.00). Insulin autoimmune syndrome was thus identified as the cause of the hypoglycemia.

The patient has continued to have hypoglycemic episodes, albeit less frequent and less severe. His insulin (2479 μIU/ml) and C peptide (4.9 ng/ml) serum levels have continued to be high, although lower than the initial levels. Because of his poor general health, we have decided not to submit him to any new invasive procedure. The patient is maintained on a hypercaloric diet and without medication.

## DISCUSSION

The majority of IAS cases are reported in Japanese subjects.^1,2^ IAS seems to be related to specific HLA class II alleles, which are 10 to 30 times more prevalent in Japanese and Koreans than in Caucasians.^2^ Nevertheless, IAS has also been reported in non-Japanese subjects. Up to 1999, ten cases had been identified in non-Japanese East Asians and only two in Hispanic subjects.^1^ Recently, a few other cases have been reported in Europe, including two Portuguese subjects.^3^ To the best of our knowledge, this is probably the first case of IAS in a Brazilian patient.

IAS is usually related to previous exposure to drugs. In a Japanese case series, almost 70% of the patients reported previous exposure to methimazole (49%), α-mercaptopropionyl glycine (43%), or glutathione (8%). Thus, drug-induced insulin antibodies are thought to be associated with drugs containing the sulfhydryl group. Moreover, Graves’ disease in particular seems to be a risk factor for insulin autoimmunity.^1^ Although it is not possible to determine whether the use of antibiotics during hospitalization triggered the autoimmunity in our case, we feel that this is probably the most plausible hypothesis. Indeed, antibiotics have already been associated with the development of IAS.^3^

Insulin levels in IAS are usually extremely high. These results, however, are a consequence of the insulin antibodies, which interfere with the insulin radioimmunoassay (RIA). If a double antibody technique is employed (using RIA), the insulin antibodies will increase immunoreactive insulin by competing with anti-insulin antibodies.^2^ On the other hand, an immunoradiometric assay (IRMA) may have lower false-positive rates and, therefore, be more reliable.^2^ Hypoglycemia is supposed to be caused by the binding and release of the insulin from the antibodies, which occur out of synchrony with the prevailing glucose concentration.^2^

Surgery is not currently indicated for the treatment of IAS. More than 80% of the patients present remission within less than three months after drug withdrawal. Steroids, plasmapheresis, and immunosuppressants can also be used in some patients. The current recommendations are to give six or more small meals and to avoid sweets except at the time of hypoglycemic attack.^1,2^ Our patient did not present clinical remission of the disease after approximately five months of symptoms. Also, steroids and verapamil were tried without success. Finally, the presence of sepsis that was resistant to several antibiotics contraindicated the use of high dose immunosuppressants. Hence, the clinical and surgical staff decided to submit him to subtotal pancreatectomy. Fortunately, the surgery was successful and the patient is free of hypoglycemic episodes.

The effects of insulin autoimmunity on the pancreas are only poorly known. Surgery has only been performed on seven patients. In three cases, histopathological examination identified hyperplasia of beta cells.^1^ One can only speculate that the presence of the disease over a long period might have induced beta cell proliferation (also suggested by the increased C peptide levels). Therefore, clinical treatment might not be as efficient as in patients with rapid remission of the disease.

Insulinoma is the most prevalent cause of hyperinsulinemic hypoglycemia.^1,4^ There-fore, primary investigation of hypoglycemia is always focused on its localization. When no tumor is located by any means available, there is always a question to be raised: is there a tumor anywhere else or is there any other cause for the hypoglycemia? Insulin antibody measurement must be performed on all hypoglycemic patients. Although quite uncommon, IAS is a cause of hypoglycemia that can be cured without surgery in the majority of the cases. Therefore, careful investigation of the autoimmune mechanisms may prevent an unnecessary surgical procedure on a hypoglycemic patient.
